# *STEAP1* Regulates Tumorigenesis and Chemoresistance During Peritoneal Metastasis of Gastric Cancer

**DOI:** 10.3389/fphys.2018.01132

**Published:** 2018-08-21

**Authors:** Yuan-Yu Wu, Jun-Nan Jiang, Xue-Dong Fang, Fu-Jian Ji

**Affiliations:** Department of Gastrointestinal Colorectal and Anal Surgery, China-Japan Union Hospital of Jilin University, Changchun, China

**Keywords:** *STEAP1*, tumorigenesis, chemoresistance, gastric cancer, peritoneal metastasis

## Abstract

In China, majority of the mortality in gastric cancer are associated with peritoneal metastasis. Since most gastric tumors are metastatic at initial diagnosis, the treatment of gastric cancer is limited to radical resection. Therefore, it is imperative to identify diagnostic and prognostic biomarkers. From 2014 to 2015, 20 patients were enrolled in the study. To search translationally upregulated genes in the context of epithelial to mesenchymal transition (EMT), polysome profiling was performed. The MTT, migration, and invasion assay were conducted to determine cell proliferation, migration, and invasion ability respectively. Experiments of gain and loss of function were performed using the overexpression plasmid, siRNA, and shRNA. Xenograft assay was established using nude mice to explore the role of targets translationally upregulated gene *in vivo*. Polysome profiling defined the landscape of translationally regulated gene products with differential expression between non-metastatic and metastatic cohorts. Six-transmembrane epithelial antigen of the prostate 1 (*STEAP1*) was found to be the most translationally upregulated gene product in either experimental groups. *STEAP1* was found to be required for cell proliferation, *in vitro* migration and invasion, and *in vivo* tumorigenesis. RNAi-mediated silencing of *STEAP1* potentiated chemosensitivity of the MKN45 cells to docetaxel treatment, highlighting the importance of *STEAP1* as a novel biomarker in gastric cancer patients with peritoneal metastasis. *STEAP1* is thus induced translationally and its expression promotes proliferation, migration, invasiveness, and tumorigenicity of gastric cancer. *STEAP1* can be a potent candidate for designing of targeted therapy.

## Introduction

In China, 300,000 patients are projected to die annually from gastric cancer (Chen et al., 2013). An alarming 50% of this mortality is associated with peritoneal metastasis (Cho et al., 2014). Radical resection remains the standard mode of management, but it cannot prevent peritoneal metastasis (Boku et al., 1990; Tanaka et al., 2000; Yoo et al., 2000; Hippo et al., 2001). Like most other tumorigenic conditions, discovery and validation of both diagnostic and prognostic markers are thus important for optimal management of gastric cancer patients with peritoneal metastasis (Poste and Fidler, 1980; Crosby et al., 2001).

Previous work have shown that legumain might be an important marker since it is universally overexpressed in metastatic gastric cancer patients (Dando et al., 1999; Barrett et al., 2001; Guo et al., 2013; Li et al., 2013; Zhang et al., 2016). In addition, our earlier work has shown that the RNA binding protein, poly r(C) binding protein 1 (PCBP1)-mediated regulation of microRNA-3978 (miR-3978) inhibits the expression of legumain in normal peritoneum (Zhang et al., 2016; Ji et al., 2017). Expression of both PCBP1 and miR-3978 is downregulated whereas expression of legumain is upregulated in gastric cancer patients with peritoneal metastasis (Zhang et al., 2016; Ji et al., 2017). Legumain can potentiate metastatic progression by proteolytic activation of other zymogens or by augmenting epithelial to mesenchymal transition (EMT) via activation of Akt and MAPK signaling pathways (Murthy et al., 2005; Bajjuri et al., 2011; Zhen et al., 2015; Cui et al., 2016).

In ovarian, pancreatic, and breast cancer, PCBP1 can regulate the pro-oncogenic p63 transcript stability (Cho et al., 2013). In addition, suppression of PCBP1 expression or post-translational modification can increase the translation of genes and long non-coding RNAs (LncRNAs) which were required for EMT and metastasis in different cancers, including gastric cancer (Chaudhury et al., 2010; Wang et al., 2010; Hussey et al., 2011; Liu et al., 2015; Zhang et al., 2015). In addition, inactivating mutations in PCBP1 has been identified in Burkitt lymphoma (Wagener et al., 2015). Thus it might be possible that downregulation of PCBP1 represents a central point, inhibition of which is a common mechanism to increase stemness and mesenchymal in a context dependent mode (Chaudhury et al., 2010; Wang et al., 2010; Hussey et al., 2011; Cho et al., 2013; Liu et al., 2015; Wagener et al., 2015; Zhang et al., 2015; Grelet et al., 2017; Howley and Howe, 2018). Cumulatively, this indicates that EMT might be an important part of the puzzle that regulates both peritoneal metastases in gastric cancer patients as well as induction of chemoresistance in this cohort of patients.

However, not much is known beyond expression pattern of legumain and PCBP1 during peritoneal metastasis of gastric cancer patients. And given that expression of legumain is post-transcriptionally regulated by miR-3978 and loss of PCBP1 is known to translationally induce gene products that are EMT facilitators, our objective in the current study was to define the post transcriptional landscape of EMT regulators in gastric cancer patients with peritoneal metastasis. Our analysis revealed that the membrane bound mesenchymal cell marker, six-transmembrane epithelial antigen of the prostate 1 (*STEAP1*) is translationally induced during peritoneal metastasis and can potentially drive both tumorigenesis and chemoresistance to docetaxel.

## Materials and Methods

### Patient Samples

From 2014 to 2015, 20 patients were enrolled in the current study. These patients received surgery because of gastric cancer at the China-Japan Union Hospital of Jilin University. There were 12 male and 8 female patients. The age of the 20 patients ranged from 39 to 78 years (mean age was 61.34 years). The inclusion criteria were presence of peritoneal metastasis at the time of initial presentation, which was independently confirmed by two pathologists. Tumor tissue and tumor-adjacent normal gastric tissue samples were obtained from all patients during surgery. Ethics Committee of the China-Japan Union Hospital of Jilin University approved the protocol of the study. Written informed consents were obtained from all patients.

### Cell Culture and Treatment

The HMrSV5 and MKN45 cell lines were obtained from BeNa Culture Collection (Beijing, China). The cells were cultured using RPMI 1640 medium (Life Technologies, Shanghai, China) containing 20% FBS (Lonza, Germany) and maintained in a 37°C incubator with 5% CO_2_. Where indicated cells were treated with indicated dose of docetaxel.

### Plasmids and Transfection

*STEAP1* overexpression plasmid, siRNA, and shRNA were obtained from Open Biosystems. The indicated cells were either transfected with *STEAP1* plasmids or *Firefly luciferase* overexpression constructs, or siRNAs targeting either *STEAP1* or *Renilla luciferase* using Lipofectamine 3000 (Life Technologies, Shanghai, China).

### Polysome Profiling

Cells and tissue samples were treated with cycloheximide (50 μg/mL; Sigma-Aldrich, Shanghai, China) for 30 min at 37°C. Then the cells were washed by cold PBS which contained 50 μg/mL cycloheximide. Cells were lysed and processed for polysome profiling as described before (Chaudhury et al., 2010).

### RNA Isolation From Polysomal Fractions and Quantitative Real Time Polymerase Chain Reaction (qRT-PCR)

The RNA from the various polysome fraction and total lysate aliquots were extracted using the TRIzol LS reagent (Life Technologies, Shanghai, China) according to the instruction of manufacturers. Complementary DNA (cDNA) were generated from the RNA samples in order to act as the template of the Human EMT RT^2^ Profiler PCR Array (Qiagen, Beijing, China) which contained 84 key genes that either change their expression during EMT or regulate the expression of those genes. Data was normalized to *GAPDH* expression.

### MTT Assay

To measure the rates of cell proliferation, the experiment was performed using the MTT assay kit (Sigma-Aldrich, Shanghai, China). Cells (10^6^) were seeded in triplicates on day 0 and proliferation ere measured after 24, 48, and 72 h, respectively. The relative optical density (OD) was measured at 570 nm and data was represented as mean ± standard deviation from at least three independent experiments.

### Cells Migration and Invasion Assays

The analyzes of cell migration and invasion ability were performed using cell migration assay kits (R&D Systems) following manufacturer’s protocols. BME was used as the matrix in the invasion assay.

### Xenograft Assay

SPF grade male six-week old BALB/c nude mice were purchased from the Charles River Laboratories (Beijing, China). The animal experiments were proved by the Institute’s Animal Care and Use Committee., the parental MKN45 cells or MKN45 cells which were stably transfected with two different shRNAs targeting *STEAP1* (shRNA-1 and shRNA-2) were used to establish the tumor model (*n* = 4 in each group) as previously described (Zhang et al., 2016). Cells stably expressed *Firefly luciferase* to aid in the *in vivo* luciferase imaging to track tumor formation over indicated time frame. Weights of animals were measured on alternative days until the end of the experiment.

### Statistical Analyses

SPSS statistical software program 18.0 (IBM Corporation, NY, United States) was chosen to conduct the data analyzes. A *p*-value < 0.05 was considered as statistically significant.

## Results

**FIGURE 1:**
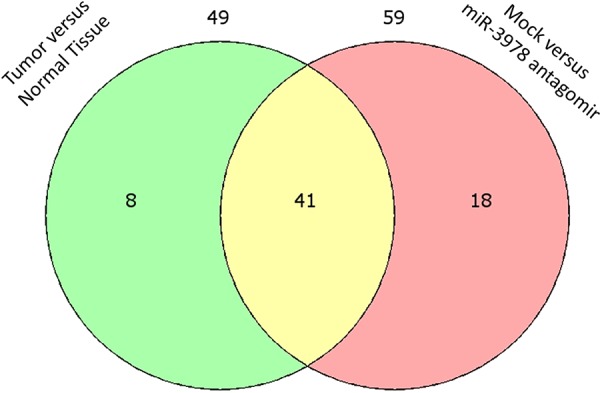
Differential translation of EMT effector genes in metastatic gastric cancer. Venn diagram showing overlap of differentially translated genes when peritoneal gastric cancer and adjacent normal control tissue specimens were compared to differential translation of same genes in mock transfected or miR-3978 antagomir transfected normal human mesothelial cell line HMrSV5 (also refer **Supplementary Tables S1**, **S2**).

Since we earlier showed that legumain expression is post-transcriptionally downregulated by miR-3978 in normal peritoneum and is deregulated during metastatic progression (Zhang et al., 2016), we were interested in defining the post-transcriptional landscape during peritoneal metastasis progression of gastric cancer. Legumain has been shown to promote mesenchymal markers in these patients (Zhang et al., 2016). Hence, we performed polysome profiling to investigate the differentially translated genes in the context of EMT. Tissue specimens from peritoneal metastasis patients and tumor-adjacent normal controls were subjected to polysome profiling. We have earlier shown that miR-3978 expression is high in the normal human mesothelial cell line, HMrSV5, whereas it is low in well-differentiated cell line mimicking peritoneal metastasis, MKN45 (Zhang et al., 2016; Ji et al., 2017). To understand the EMT effector genes that are potentially being impacted by the expression of miR-3978, we also performed polysome profiling in the HMrSV5 cells, either mock transfected or transfected with anti-miR-3978 antagomir. HMrSV5 cells transfected with anti-miR-3978 antagomir has been previously shown to mimic properties of the highly metastatic MKN45 cells (Zhang et al., 2016). Total RNA was isolated from non-translating low molecular weight non-polysomal fractions and actively-translating high molecular weight polysome fractions from triplicate set of tissue specimens or cell lines and then used to template a qRT-PCR reaction using the Human EMT RT^2^ Profiler PCR Array that comprised of 84 key genes which either can change their expression during EMT or regulate the expression of those genes (**Supplementary Tables S1**, **S2**). Of the 49 differentially translated genes in the tumor versus normal tissue control and the 59 differentially translated genes in the mock treated versus anti-miR-3978 antagomir treated MKN45 cell, 41 were common (**Figure 1**), indicating the similarity between the two chosen experimental models. Well-differentiated cell line mimicking peritoneal metastasis, MKN45, and the human mesothelial cell line, HMrSV5.

Among the differentially translated genes in both groups, *STEAP1* (encoding six-transmembrane epithelial antigen of prostate) was the most enriched in the polysome. Polysome profiling results were validated by immunoblotting for *STEAP1* (data not shown).

**FIGURE 2:**
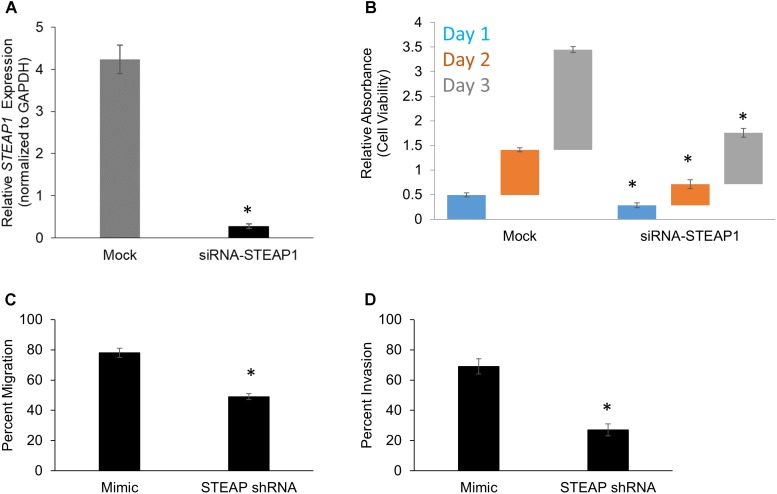
*STEAP* potentiates cell growth and *in vitro* metastatic properties in gastric cancer. **(A)** *STEAP1* mRNA expression was significantly downregulated in MKN45 cells transfected with siRNA targeting *STEAP1*. Relative mRNA expression of *STEAP1*, normalized to *GAPDH* expression, in mock and siRNA-*STEAP1* are shown. Data represents mean ± standard deviation of three independent replicates. MTT assay-based determination of cell proliferation **(B)**, *in vitro* migration **(C)**, and *in vitro* invasion **(D)** in mock transfected MKN45 cells and MKN45 cells transduced with siRNA targeting *STEAP1*. In each case, data shown are mean ± standard deviation of three independent replicates. ^∗^*p* < 0.05.

We next wanted to determine the role of *STEAP1* in the pathogenesis of peritoneal metastasis. To determine effect of *STEAP1* expression on intrinsic changes in cell proliferation ability, the assays of cell viability were performed in mock or MKN45 cells which were transfected by siRNA targeting *STEAP1*. RNAi-mediated silencing of *STEAP1* in MKN45 cells was verified by qRT-PCR (**Figure 2A**). RNAi-mediated silencing of *STEAP1* in MKN45 cells (Mimic: Day 1 – 0.49 ± 0.04, Day 2 – 0.91 ± 0.04; Day 3 – 2.04 ± 0.06 / siRNA-*STEAP1*: Day 1 – 0.29 ± 0.05, Day 2 – 0.43 ± 0.09; Day 3 – 1.04 ± 0.09) significantly inhibited cell proliferation compare to the controls (*p* < 0.05 in each case) (**Figure 2B**).

Each of the aforementioned transfectants in MKN45 cells were also subjected to *in vitro* migration (**Figure 2C**) and invasion (**Figure 2D**) assay in standard transwell assays. Silencing of *STEAP1* expression suppressed migration (78 ± 3% versus 49 ± 2%; *p* < 0.001) and invasion (69 ± 5% versus 27 ± 4%) in MKN45 cells based on these criteria. Taken together, these results suggested that *STEAP1* expression is associated to proliferative capacity of MKN45 cells and also functions associated with mesenchymal cells, namely migration and invasion.

**FIGURE 3:**
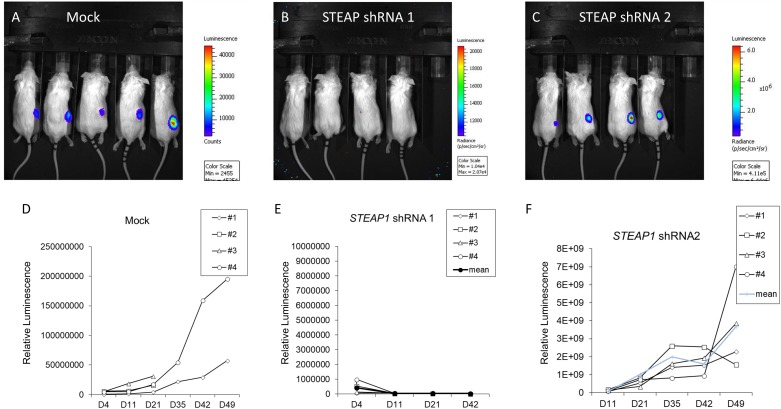
*STEAP* potentiates tumor growth *in vivo*. Panels **(A–C)** Bioluminescence images obtained in mice 6 weeks after implantation with either mock-transduced MKN45 cells, or MKN45 cells transduced with two independent shRNAs targeting *STEAP1*. Please note the intensity axis on the right of each panel. **(D–F)** Corresponding quantification of tumor growth patterns for images shown in **A–C**. ^∗^*p* < 0.05 (also refer **Supplementary Figure S1**).

Since our results indicated that *STEAP1* can dictate cell proliferation, we investigated the role of *STEAP1* expression on tumorigenesis *per se*. MKN45 cells stably expressing two independent shRNAs targeting *STEAP1* were generated and used for xenograft studies. Successful knockdown of *STEAP1* was confirmed by qRT-PCR (**Supplementary Figure S1**). shRNA-1 was significantly more potent in silencing *STEAP1* expression, compared to shRNA-2. Compared with mock transduced MKN45 cells, *STEAP1* silencing completely inhibited (shRNA-1) or significantly attenuated (shRNA-1) the growth of tumor (**Figures 3A–C**). There were no significant changes in body weights of the animals among the different experimental groups (data not shown). Tumor growth rate measured by *in vivo* luminescence showed a similar trend (**Figures 3D–F**), with shRNA-1 showing a better effect than shRNA-2. The differences observed with the two shRNAs can be attributed to the degree of *STEAP1* knockdown achieved by the respective shRNA (**Supplementary Figure S1**). Taken together, these data suggest that high expression of *STEAP1* is required for gastric cancer formation and progression.

**FIGURE 4:**
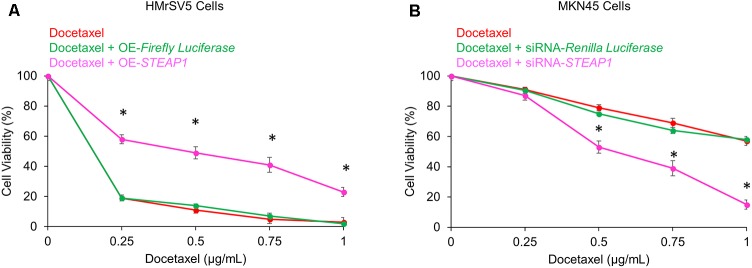
Modulation of *STEAP1* expression changes susceptibility to docetaxel treatment. **(A)** *Firefly luciferase* or *STEAP1* overexpressing HMrSV5 cells were treated with indicated doses of docetaxel 72 h. **(B)** MKN45 cells transiently transfected with siRNA targeting *Renilla luciferase* or *STEAP1* were treated with indicated doses of docetaxel for 72 h. MTT assay was performed to assess the cell viability after 72 h. Data was obtained from three independent replicates experiments and was represented as mean ± standard deviation (SD). ^∗^*p* < 0.05.

Docetaxel-based therapy has emerged as the treatment of choice in gastric cancer patients (Tetzlaff et al., 2008). Thus, to explore the potential effects of *STEAP1* expression on chemosensitivity to docetaxel we either overexpressed *STEAP1* in the mesothelial cell line HMrSV5 or silenced its expression in the metastatic MKN45 cells before treatment with docetaxel. Overexpression of *STEAP1* made HMrSV5 cells significantly resistant to docetaxel treatment (*p* < 0.05) (**Figure 4A**). Conversely, downregulation of *STEAP1* significantly increased chemosensitivity in MKN45 cells to docetaxel treatment (*p* < 0.05) (**Figure 4B**).

## Discussion

The profound induction in relative expression of *STEAP1* by translational regulatory mechanisms in metastatic gastric cancer patients along with its capacity to promote mesenchymal traits and chemoresistance *in vitro* and tumorigenesis *in vivo* suggest that *STEAP1* is a critical determinant that drives EMT like programs during peritoneal metastasis of gastric cancer.

Our findings corroborate recently reported finding that *STEAP1* potentiates oxidative properties and invasiveness in Ewing tumor cells (Grunewald et al., 2012). *STEAP1* encodes for the six-transmembrane epithelial antigen of the prostate 1, which is a membrane-bound protein functioning in mitochondrial transmembrane electron transfer (Hubert et al., 1999; Sendamarai et al., 2008). *STEAP1* is overexpressed in wide variety of cancers including prostate and bladder cancer (Hubert et al., 1999; Ohgami et al., 2006; Moreaux et al., 2012). *STEAP1* has also been classified as a mesenchymal stem cell marker (Vaghjiani et al., 2009), supporting the relationship we observed with peritoneal metastasis of gastric cancer.

*STEAP1* mRNA is hardly expressed in benign tissues (Hubert et al., 1999; Valenti et al., 2009). Our results here show that *STEAP1* expression is upregulated at the translational level. Thus, given its high tumor specificity and membrane-bound localization, *STEAP1* is an attractive candidate for targeted therapy in gastric cancer patients with peritoneal metastasis (Hubert et al., 1999; Cheung et al., 2007; Valenti et al., 2009). In fact, it has been shown that monoclonal antibodies designed against *STEAP1* can inhibit bladder and prostate cancer in mice models (Challita-Eid et al., 2007). It will be interesting and vital to determine if these or similar monoclonal antibodies will be able to prevent onset and progression of gastric cancer.

In summary, we have shown that in the present study the putative oncogenic and pro-metastatic function of *STEAP1*. We prove that *STEAP1* is induced translationally and that its expression promotes proliferation, migration, invasiveness, and tumorigenicity of gastric cancer. The regulatory mechanisms dictating translational upregulation of *STEAP1* and the benefit of targeted therapy against *STEAP1* alone and in the context of chemoresistance in patients with peritoneal metastasis will be extensive areas of future investigation.

## Author Contributions

Y-YW conceived and designed the study, and acquired the data. J-NJ analyzed and interpreted the data, and drafted the manuscript. X-DF revised the manuscript for intellectual content. F-JJ approved the final manuscript.

## Conflict of Interest Statement

The authors declare that the research was conducted in the absence of any commercial or financial relationships that could be construed as a potential conflict of interest.

## Supplementary Material

FIGURE S1 Efficiency of shRNA-1 and 2 targeting *STEAP1* to downregulate *STEAP1* expression. Relative mRNA expression of *STEAP1*, normalized to *GAPDH* expression, in parental MKN45 cells and MK N45 cells stably transduced with two different shRNAs (shRNA-1 and shRNA-2) targeting *STEAP1*. Data represents mean ± standard deviation of three independent replicates. ^∗^*p* < 0.05.

TABLE S1 Genes showing 2-fold changes in polysome occupancy between tumor and tumor adjacent normal gastric cancer tissue with peritoneal metastasis.

TABLE S2 Genes showing 2-fold changes in polysome occupancy in the normal human mesothelial cell line, HMrSV5, either mock transfected or transfected with anti-miR-3978 antagomir.
