# Supplementary figures and images for: STEAP1 Regulates Tumorigenesis and Chemoresistance During Peritoneal Metastasis of Gastric Cancer
Source: Front Physiol. 2018 Aug 21;9:1132. doi: 10.3389/fphys.2018.01132 (PMC6110897; doi:10.3389/fphys.2018.01132)

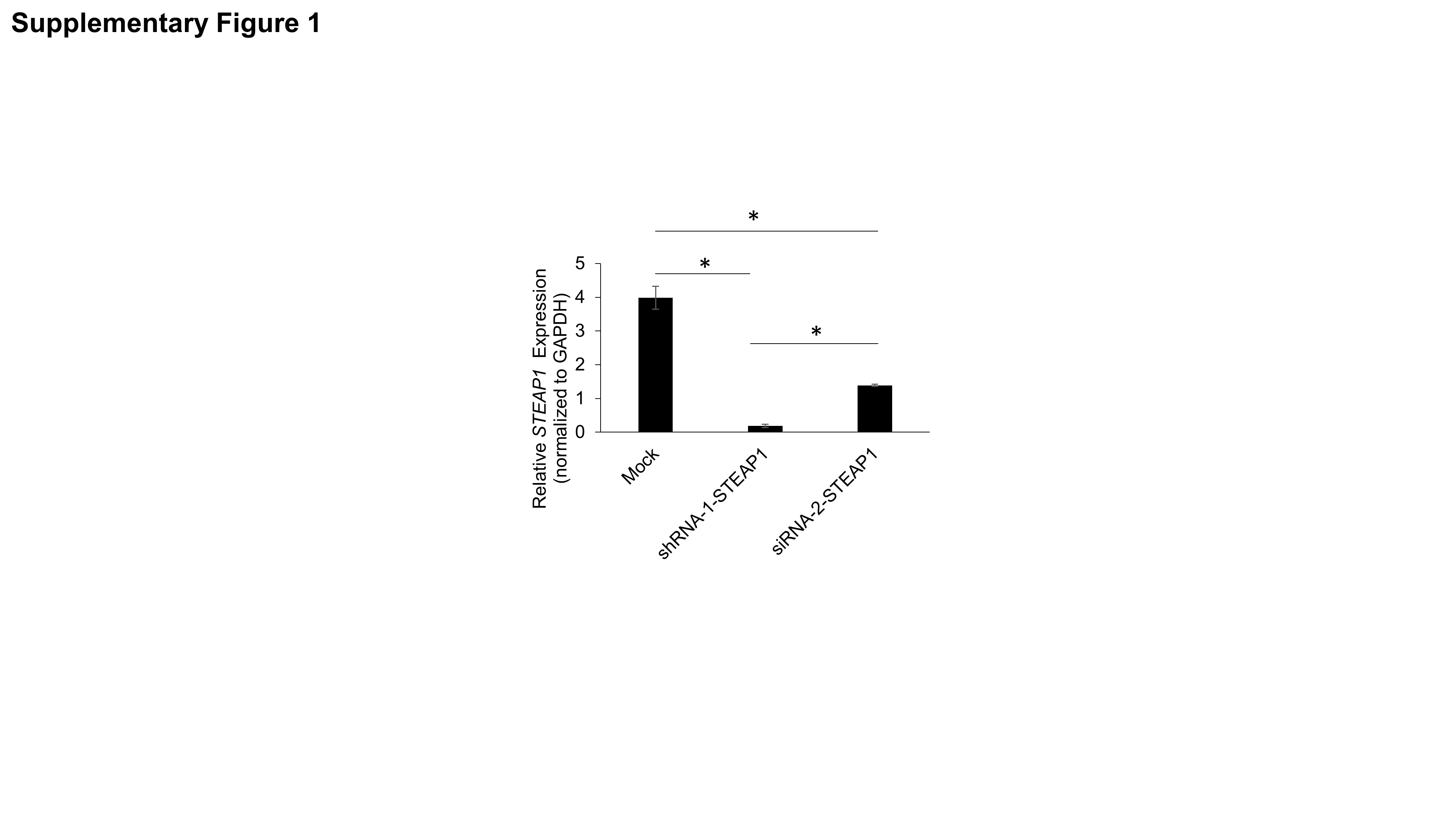

Supplement: FIGURE S1 — Efficiency of shRNA-1 and 2 targeting STEAP1 to downregulate STEAP1 expression. Relative mRNA expression of STEAP1, normalized to GAPDH expression, in parental MKN45 cells and MK N45 cells stably transduced with two different shRNAs (shRNA-1 and shRNA-2) targeting STEAP1. Data represents mean ± standard deviation of three independent replicates. ∗p < 0.05. [file Image_1.TIF]
