# Supplementary material for: STEAP1 Regulates Tumorigenesis and Chemoresistance During Peritoneal Metastasis of Gastric Cancer
Source: Front Physiol. 2018 Aug 21;9:1132. doi: 10.3389/fphys.2018.01132 (PMC6110897; doi:10.3389/fphys.2018.01132)
Supplement: TABLE S1 — Genes showing 2-fold changes in polysome occupancy between tumor and tumor adjacent normal gastric cancer tissue with peritoneal metastasis. [file Table_1.docx]

Supplementary Table 1: Genes showing 2-fold changes in polysome occupancy between tumor and tumor adjacent normal gastric cancer tissue with peritoneal metastasis.

| Target | ΔΔCt -(dGOI-dACTB) |
| --- | --- |
| CDH1 | -13.58676555 |
| KRT14 | -13.44893051 |
| CALD1 | -10.1317101 |
| FGFBP1 | -8.883012507 |
| EGFR | -8.662131156 |
| CAV2 | -8.117704437 |
| IL1RN | -8.04594562 |
| SPARC | -7.402060111 |
| GNG11 | -7.296916988 |
| DSP | -7.142164005 |
| JAG1 | -6.59340697 |
| CAMK2N1 | -6.082348211 |
| ERBB3 | -5.410231723 |
| OCLN | -3.019108176 |
| GSC | -2.971947759 |
| SNAI2 | -2.833694043 |
| IGFBP4 | -2.654389293 |
| F11R | -2.385828188 |
| COL3A1 | -2.02909923 |
| RGS2 | 2.030349063 |
| ITGB1 | 2.09488092 |
| MSN | 2.161085002 |
| HPRT1 | 2.371853484 |
| SNAI3 | 2.374755364 |
| SMAD2 | 2.482461689 |
| NUDT13 | 2.606056781 |
| RAC1 | 2.7460407 |
| PTP4A1 | 2.779108191 |
| TGFB1 | 3.028674307 |
| DSC2 | 3.200758196 |
| GUSB | 3.210883741 |
| ILK | 3.242280261 |
| GSK3B | 3.257168712 |
| VIM | 4.106523109 |
| TIMP1 | 4.30076343 |
| SERPINE1 | 4.341908564 |
| TSPAN13 | 4.598832574 |
| PLEK2 | 4.712521881 |
| TCF4 | 4.897236191 |
| MMP9 | 4.948177513 |
| ZEB2 | 5.171783005 |
| ESR1 | 6.118716717 |
| TBP | 6.356924105 |
| ZEB1 | 6.476895789 |
| BMP1 | 6.623531269 |
| MMP2 | 6.662879625 |
| VPS13A | 7.318973134 |
| SPP1 | 9.176997028 |
| STEAP1 | 13.58333641 |
