# Supplementary material for: STEAP1 Regulates Tumorigenesis and Chemoresistance During Peritoneal Metastasis of Gastric Cancer
Source: Front Physiol. 2018 Aug 21;9:1132. doi: 10.3389/fphys.2018.01132 (PMC6110897; doi:10.3389/fphys.2018.01132)
Supplement: TABLE S2 — Genes showing 2-fold changes in polysome occupancy in the normal human mesothelial cell line, HMrSV5, either mock transfected or transfected with anti-miR-3978 antagomir. [file Table_2.docx]

Supplementary Table 2: Genes showing 2-fold changes in polysome occupancy in the normal human mesothelial cell line, HMrSV5, either mock transfected or transfected with anti-miR-3978 antagomir.

| Target | ΔΔCt-(dGOI-dACTB) |
| --- | --- |
| KRT14 | -14.70909846 |
| CDH1 | -10.16403222 |
| FGFBP1 | -10.14318046 |
| EGFR | -9.922299106 |
| CAV2 | -9.377872387 |
| CAMK2N1 | -6.812881093 |
| ERBB3 | -6.670399674 |
| CALD1 | -5.603340404 |
| SNAI2 | -4.093861994 |
| SNAI1 | -3.365692458 |
| BMP7 | -3.152580076 |
| MST1R | -3.089601257 |
| OCLN | -3.019108176 |
| TGFB2 | -2.910035027 |
| JAG1 | -2.44038475 |
| GNG11 | -2.416982126 |
| MAP1B | -2.055701253 |
| B2M | 2.005312369 |
| PTK2 | 2.079007825 |
| FZD7 | 2.409042331 |
| SMAD2 | 2.428352632 |
| MSN | 2.489250069 |
| GSK3B | 2.668609481 |
| IGFBP4 | 2.703054025 |
| HPRT1 | 2.734656107 |
| ITGB1 | 2.798672833 |
| PTP4A1 | 2.854646255 |
| NUDT13 | 2.973643992 |
| GUSB | 3.000308977 |
| SNAI3 | 3.032419571 |
| RAC1 | 3.158302133 |
| COL1A2 | 3.23255194 |
| VIM | 3.305567993 |
| TSPAN13 | 3.305915275 |
| ITGAV | 3.400353798 |
| AKT1 | 3.405153107 |
| DSC2 | 3.406898584 |
| PPPDE2 | 3.42140055 |
| NOTCH1 | 3.512699858 |
| TIMP1 | 3.567722687 |
| SERPINE1 | 3.653192369 |
| BMP1 | 3.89644164 |
| ZEB1 | 3.939048743 |
| TCF4 | 4.089849208 |
| COL3A1 | 4.298231334 |
| TBP | 4.494258352 |
| ESR1 | 4.654890298 |
| TGFB1 | 4.659439325 |
| PLEK2 | 4.750615852 |
| MMP9 | 5.483921798 |
| ZEB2 | 5.69514809 |
| TGFB3 | 7.17912015 |
| TMEM132A | 7.221387789 |
| FN1 | 7.741973757 |
| KRT19 | 8.131154923 |
| VPS13A | 9.53981728 |
| SPP1 | 10.50717394 |
| VCAN | 10.62851699 |
| STEAP1 | 13.40241666 |
